# Opioid substitution therapy for people living in German prisons—inequality compared with civic sector

**DOI:** 10.1186/s12954-019-0340-4

**Published:** 2019-12-21

**Authors:** Heino Stöver, Daniela Jamin, Ingo Ilja Michels, Bärbel Knorr, Karlheinz Keppler, Daniel Deimel

**Affiliations:** 1 Fachbereich 4: Soziale Arbeit und Gesundheit, Faculty “Health and Social Work”, Institute for Addiction Research, Frankfurt University of Applied Sciences, Nibelungenplatz 1, 60318 Frankfurt, Germany; 2 Fachbereich 4: Soziale Arbeit und Gesundheit, Faculty “Health and Social Work”, Frankfurt University of Applied Sciences, Nibelungenplatz 1, 60318 Frankfurt, Germany; 3 Fachbereich 4: Soziale Arbeit und Gesundheit, Faculty “Health and Social Work”, Institute for Addiction Research, Frankfurt University of Applied Sciences, Nibelungenplatz 1, 60318 Frankfurt, Germany; 4 Deutsche AIDS-Hilfe, Wilhelmstr. 138, 10963 Berlin, Germany; 5 Berlin, Germany; 6 Department Aachen, German Institute of Addiction and Prevention Research, Catholic University of Applied Science North Rhine-Westphalia, Robert-Schumann-Str. 25, 52044 Aachen, Germany

**Keywords:** Opioid substitution therapy, Health inequality, Imprisonment, Incarceration, Methadone

## Abstract

**Background:**

The above-average proportion of people with opioid use disorder living in prisons is a worldwide reality, and the need to treat these people was recognized internationally more than 20 years ago. Studies have shown that substitution therapies are best suited to treat opioid use disorder and reduce the risk of HIV and hepatitis C transmission and overdose. However, huge health inequalities exist in and outside of prison due to the different implementation of opioid substitution therapy (OST). People living in prisons are entitled to the best possible health care. This is established by the Universal Declaration of Human Rights and by the International Convention on Economic, Social and Cultural Rights. Solely the imprisonment, and not the loss of fundamental human rights, constitutes the punishment.

**Methods:**

A qualitative literature search using PubMed and Google Scholar was performed in order to identify relevant publications.

**Results:**

This review shows the inequality in availability of opioid substitution therapy for people living in prison compared with people outside of prison in Germany. It also gives possible reasons and evidence for this inequality, showing that continuing or initiating OST in prison is more beneficial for the health of people living in prison than abstinence-oriented treatment only.

**Conclusion:**

It is important that drug use disorder is treated as a serious illness also in prison. Joint efforts are needed to provide people living in prison with the best possible treatment and to minimize the adverse effects of drug use. Therefore, with laws, policies, and programs that conform to international human rights standards, each state must ensure that people living in prison receive the same health care as people outside of prison.

## Background

The criminalization of drug use leads to an over-representation of opioid use disorder in people living prison [1]. Opioid use disorder is often associated with fatal consequences, especially for people living in prison. These consequences include an increased risk of hepatitis B and C, and HIV due to the use of contaminated syringes, and an above-average mortality rate shortly after release from prison, mostly as a result of fatal overdose [2]. In Europe, Asia, and North America, about one-third of the total male prison population has opioid use disorder—in some Central Asian countries even up to 80% [3, 4]—while in women’s prison more than 50% have opioid use disorder [5]. The different forms of treatment for opioid use disorder mainly fall into two categories: opioid substitution therapies with complementary psychosocial care and abstinence programs. A substitution therapy is the medically supervised administration of a prescribed substance, which is pharmaceutically related to the substance on which a person is dependent. Examples are insulin treatment in diabetic patients or opioid substitution therapy (OST) in people with opioid use disorder. The prescribed opioid agonists used for OST have effects on the central nervous system (CNS) similar or identical to heroin or morphine, alleviating withdrawal symptoms and inhibiting the desire for prohibited opioids [6]. Dole and Nyswander carried out the first substitution therapy in prison in the 1960s [7, 8].

Subsequently, OST became an established treatment for people outside of prison but not for people living in prison. The need for OST to treat people with opioid use disorder living in prisons was internationally endorsed in 1993 and confirmed by UN agencies including the United Nations Office on Drugs and Crime (UNODC), the World Health Organization (WHO), and others in 2013: “Considering that opioid substitution therapy is the most effective drug dependence treatment for people dependent on opiates, where it is available in the community, it should be accessible in prisons.” [9]. Primary rules for the health care of people living in prison at the political level are the principle of equivalence (established by the United Nations, UN) [10], the European Prison Rules (revision of the minimum principles for the treatment of people living in prison), and the technical level the declarations of the World Medical Association. More and more prison systems worldwide are offering substitution programs for people living in prison. Examples include Australia and Canada, some US states, most countries of the European Union, and a number of other countries such as Indonesia or the Islamic Republic of Iran [4]. However, huge health inequalities due to the different implementation of OST in prison and outside of prison still exist [11]. For example, OST is nominally available in German prisons, but its implementation depends on the federal state, prison, and prison doctors. In addition, there is hardly any psychosocial care in prisons, and if there is, then only in a reduced form and with months of waiting time [12].

One of the best-known court cases in Germany where substitution therapy was refused in prison is that of “Wenner versus Germany” [13]:

*Mr. Wenner (born in 1955) had been using heroin for 17 years. From 1991–2008 he received treatment with methadone and benefited significantly from this therapy, but subsequently resumed his illicit drug use. After being sentenced to 6 years imprisonment for drug trafficking to finance his own consumption, he asked for the resumption of substitution therapy in prison. The Bavarian judicial authorities and courts refused and instead ordered treatment based exclusively on abstinence. This decision turned out to be a mistake because Mr. Wenner subsequently consumed a number of psychoactive substances available on the black market of the prison. He continued to demand methadone. Because his request was still denied, he demanded his health status and treatment to be assessed by external specialists. This was also rejected. It was not until the end of 2014, when Mr. Wenner was released from prison, that his methadone treatment was resumed. Mr. Wenner lodged his appeal with the Court, arguing that the two refusals he had received during his imprisonment infringed Article 3 of the European Convention on Human Rights (ECHR). In its judgment of 1 September 2016, the European Court of Human Rights ruled that, indeed, the refusal to provide an indicated substitution therapy during the prison sentence violates Art. 3 of the ECHR. In particular, the prison should have consulted independent experts.*

In the case of Mr. Wenner, who had received a successfully functioning OST for more than 16 years, the therapy should therefore have been continued in prison. Although this judgment does not prejudge a medical decision for each individual case, it emphasizes the principle of equivalence and defines the lack of (external) assessment or appraisal despite different assessments of the indication of substitution therapy as a human rights violation.

The court case of Mr. Wenner, which is representative of many others, raises a fundamental question: Why is the care for people with drug use disorder living in prison not equivalent to the care provided to people outside of prison? This question is all the more important since there is a plethora of such social inequalities. For example, people living in prison and preventive detention in Germany are generally not covered by health and long-term care insurance, although they are obliged to work and many of these people do work. In addition, people living in prison do not have a free choice of doctor. Outside the prison, a patient may change doctors or report a violation of the regulations to the Medical Association. This review illustrates the different acceptance of OST in prisons in the various German federal states on the basis of surveyed data and deals with the reasons for the unequal treatment compared with people outside of prison. Moreover, this review provides evidence in favor of equal treatment of people outside of prison and people living in prison.

## Methods

We performed a qualitative literature search using PubMed and Google Scholar in order to identify relevant publications. The number of people with opioid use disorder living in German federal state prisons who receive OST was obtained from official prison data [10, 14–17], Deutsche AIDS-Hilfe [12], and our own surveys. However, currently, there is no comprehensive overview for Germany as not all federal states publish their data.

## Results and discussion

### Substitution therapy of opioid use disorder

Opioid use disorder is a serious chronic disease. Usually, it requires lifelong treatment in which physical, psychological, and social aspects must be considered equally [18, 19]. Substitution treatment is an evidence-based and scientifically well-evaluated form of therapy and represents the therapy of choice for the majority of patients. The aims of substitution therapy include ensuring survival, stabilizing and improving the state of health, reducing the use of other substances, and improving the health-related quality of life [12]. The characteristics of good substitution therapy include a patient-specific approach, continuous treatment application, adjustment to the treatment of other chronic diseases, e.g., an anti(retro)viral therapy, management of relapse risk, and regular monitoring of patients.

#### Substances used for OST

For OST, opioid agonists are mainly used, including methadone, levomethadone, buprenorphine (mixed agonist/antagonist), retarded morphine, diamorphine, and codeine [20, 21].

The most effective treatment for the opioid use disorder involves maintenance therapy with the opioid agonist methadone and the mixed opioid agonist/antagonist buprenorphine [22–24], but pure antagonists such as naltrexone are also used. The latter inhibit the action of other opiates by occupying the same receptor sites in the CNS but do not stop the desire for them. If an antagonist is taken first and then an opiate, the opiate has no euphoric effect because it cannot affect the CNS. If an antagonist is taken after the opiate, symptoms of opiate withdrawal occur immediately; antagonists are therefore contraindicated if opiate detoxification has not yet taken place.

Both buprenorphine and methadone are associated with reduced in-prison risk behavior, increased post-release treatment retention, and reduced ongoing opioid use, overdose, and death [25]. When deciding whether substitution therapy is indicated, the benefits of substitution therapy must be weighed against the risks of uncontrolled drug use. Substitution treatment can also be initiated for patients with opioid use disorder who are not currently consuming—e.g., inmates with a high risk of relapse and mortality [26].

### Health inequality in German federal states

#### Health inequality

In recent years, health inequalities have become a central theme of research, reporting, and politics. Social epidemiology has been established as an independent research discipline focusing on the analysis of health inequalities [27]. Health monitoring now regularly presents data and facts on the extent and development of health inequalities. These data show, for example, that many diseases and health problems, and also behavioral health risks such as smoking and drug use, occur more frequently among people with low incomes, inadequate education, and low occupational status [28].

From a public health and health policy perspective, reducing health inequality is an important goal. Monitoring current social epidemiology developments can help to identify new or emerging health inequalities and thus also possible target groups and settings for interventions. This is all the more important because the experience of recent years has shown that many measures and interventions, especially in the field of prevention and health promotion, do not sufficiently reach the socially disadvantaged population groups [29]. Despite positive developments, such as the adoption of the Act to Strengthen Health Promotion and Prevention (Prevention Act - PrävG) [30], which came into force in 2015, and the continuity of cooperation alliances, it is clear that Germany has yet to develop a comprehensive policy strategy to reduce health inequalities. Part of this strategy must be to demonstrate and prevent health inequities, i.e., health inequalities that denote an unjust difference in health [31, 32].

#### OST in prison: situation in German federal states

In Germany, about 64,000 people are in imprisonment (reference date 31.08.2016) and about 30–40% of people living in prisons have a drug use disorder [33, 34]. Assistance for people with drug use disorder living in prison is usually limited to information, education, and abstinence-oriented treatment, and is directed towards overcoming drug dependence [35]. Although the German government has pointed out that successful substitution is the best protection against drug death in people with opioid use disorder, the German AIDS Federation estimates that only 5–9% of people with opioid use disorder living in prison receive OST [36] compared with about 48% of people outside of prison [37, 38].

The implementation of OST in the prison sector varies considerably between the individual federal states and the individual prisons. The northern federal states in particular show high OST rates whereas OST is carried out less frequently in the Saarland, Bavaria, and eastern federal states. Data on OST in prisons are available from 15 federal states (Table 1).

**Table 1:** People with opioid use disorder living in prison, who receive OST. Overview of the individual German federal states ([10, 14–17] and “Deutsche AIDS-Hilfe”)

Federal State	Year of evaluation	Number of people living in prison in the reference year	Approx. number of people with opioid disorder living in prison (reported number, or 30% of total people living in prison)	People with opioid use disorder living in prison, who receive OST, *N* (approx. %)
Bremen	2018	620	186	90–120 (48–65%)
North Rhine-Westphalia	2018	16,219	3660	2048 (56%)
Schleswig-Holstein	2018	1150	350	130–150 (37–43%)
Hamburg	2018	1900	570	150–200 (26–35%)
Hesse	2018	4600	1380	430 (31%)
Berlin	2018	3050	915	246 (27%)
Lower Saxony	2018	4750	1425	310 (22%)
Saarland	2018	765	230	27 (12%)
Rhineland-Palatinate	2018	3050	915	105 (11%)
Baden-Wuerttemberg	2018	7390	1832	168 (9%)
Sachsen-Anhalt	2018	1566	470	36 (8%)
Thuringia	2018	1500	450	30 (7%)
Bavaria	2018	11,000	3300	240 (7%)
Brandenburg	2018	1000	300	9 (3%)
Saxony	2018	3400	1020	10 (<1%)
Mecklenburg-Western Pomerania	Not available			

In Bavaria, OST is offered very rarely and then only in exceptional cases [36], for example pregnant women or seriously ill patients for whom withdrawal would aggravate the disease [36]. However, the number of OST performed in Bavarian prisons has increased in the last 2 years (source: “Deutsche AIDS-Hilfe”). Substitution therapy during prison stay is offered in all federal states, but not in all prisons. In Hesse, for example, treatment is only possible in 11 of the 16 prisons (69%) [36]. The diagnoses made outside prison are often not accepted by prison doctors. In most cases, continuous substitution is only carried out for short prison periods [36]. The number of people with treatments started outside of prison and discontinued in prison is as high as 70% [36]. A study carried out in Bavaria in 2012 even showed that almost 90% of those questioned had to abandon OST in prison [39]. Discontinuation of treatment is, according to the guidelines of the German Medical Association, only possible if the therapy proves to be unsuitable, if there is continued consumption of other hazardous substances, or if the patient repeatedly and persistently fails to adhere to agreements or violates the rules of the treating institution. All other intervention options should be exhausted before discontinuation is considered [19, 36].

In a decision by the European Court of Human rights, it was emphasized that whether withholding OST is acceptable must be examined particularly thoroughly in people who have been dependent for many years [12, 40]. This is of special importance when medical opinions and other documents (e.g., on previous failed therapies) advocating long-term substitution treatment are available [12, 40]. However, initiation of OST in prison is the exception, even in OST-practicing prisons [12].

The demand for continuing OST started by people outside of prison or for starting a substitution treatment in prison corresponds to the current state of medical research. It is also contained in the guidelines of the German Medical Association on substitution therapy in people with opioid use disorder: “In the event of a change to hospital treatment, rehabilitation measures, imprisonment, or other form of inpatient accommodation, continuity of treatment must be ensured by the institution taking over.” [19].

### Reasons for inequality of OST provision in prisons and outside of prison

The inequality in availability of OST for people living in prison compared with people outside of prison has multiple causes. For some decision makers, methadone, buprenorphine, and other substitution medications are merely mood-altering substances like any other addictive drugs, the accessibility of which delays the personal development needed for a drug-free life [20]. Sometimes, the moral objection is raised that substitution programs simply replace one addictive drug with another—an attitude that may be fueled by the term “opioid substitution therapy” [41]. In addition, the classification of people with drug use disorder living in prisons varies between “sick,” “weak in character,” and “criminal” [42]. We believe that simultaneous labeling as ill—therefore not responsible for one’s own actions—and as criminal causes situational, interactive, and executive confusion in addition to the double discrimination. For those affected, it leads to uncertainty about their own identity, resulting in difficult social relationships and a lack of ability to develop strategies for solving problems. Those affected persist in their passive indecision and have not had the chance to develop subjectively meaningful activities beyond the procurement of drugs. The interaction partners of people with substance use disorder, the prison staff, can do little, since they also have no agreement on how to deal with the problem. We observed that in everyday life, they often fluctuate between individual attribution of blame to “criminals,” “self-inflicted behavior” (“You only have to want!”), and recognition of a disease character of “drug dependence.” A 2008 survey showed that drug-free detoxification—that is, abstinence-oriented programs—is preferred by prison doctors [43]. Even today, this position still seems to be widespread for different reasons. Due to negative attitudes among doctors, the prison staff has no guidance on the use of OST [44]. Moreover, even some judges do not allow methadone because of their personal prejudices against methadone as a valid treatment [23]. In addition to these rather individual allocations, social determinants also matter: In prison as a “total institution” (named by E. Goffman [45]), functioning plays a key role and substance use disorder is often seen as disruptive to this requirement. Summarizing all these reasons, it becomes clear that the non-supply of OST in prisons is guided by ideology, morally driven arguments, and social determinants, but not by science and evidence.

Another major problem is staff scarcity and side-consumption [46]. Side-consumption, i.e., the use of other psychoactive substances that may counteract the effects of methadone or buprenorphine, is often a reason for discontinuing OST due to toxicity effects [46, 47]. In this context, a detected co-consumption should initially prompt the treating physicians to review the existing dosage, to increase the dose of the substitution administered if necessary and to draw attention to the dangers of interactions rather than to discontinue the treatment [12]. Furthermore, the reason for the co-consumption should be addressed with the help of a substance use disorder counselor, a psychologist, or a social worker. However, side-consumption is not tolerated in prisons. According to our experience, substitution would be discontinued as soon as cannabis is detected in urine. It is also not conducive that some people who are about to be imprisoned take drugs in larger quantities because they can no longer take drugs in prison.

### Why OST should be a standard therapy both in prison and outside of prison

#### Reduced use of illicit drugs and all-cause mortality

Many of the concerns raised about substitution programs have proven to be unfounded. Thus, studies have shown that the renunciation of illicit drug use and prevention of mortality are better in methadone treatment than in detoxification programs: The risk of all-cause mortality among people with opioid use disorder is 2–3 times lower when people receive opioid agonist therapy than without [48]. In an English national study, prison-based OST was associated with a 75% reduction in all-cause mortality and an 85% reduction in fatal drug-related poisoning in the first month after release from prison [49].

#### Reduced risk of relapse after release

Another study shows that between 70 and 98% of those who are imprisoned for drug offences and receive no treatment in prison relapse within 1 year of release [50, 51]. The initiation of an OST in prison could reduce this proportion: Results of a randomized clinical trial on methadone treatment of people living in prison showed that methadone therapy initiated before release from prison has short-term positive effects in terms of initiating further treatment outside of prison and reducing heroin use [52].

#### Reduced spread of HIV and hepatitis C

OST also reduces the spread of HIV and hepatitis C. Due to overcrowding, poor nutrition, inadequate precautions, continued use of illicit drugs, and unprotected sexual contacts, the risk of HIV infection in prisons is extremely high [4]. Intravenous drug users have a particularly high risk of contracting HIV and other blood-borne viruses due to sharing or reuse of injectables [53]. A number of prison surveillance studies have found HIV prevalence to be 22 times, 19 times, and 34 times higher in prisons than in surrounding communities in Ukraine, Azerbaijan, and Kyrgyzstan, respectively [54].

Interestingly, according to the DRUCK study in eight German cities, 11% of people who inject drugs started intravenous consumption in prison [55].

Intravenous drug use is the most common route of transmission of hepatitis C today [53]. Hepatitis C becomes chronic in at least 50 to 80% of all cases. In 7 to 15% of chronically infected patients, cirrhosis of the liver develops within 20 years, which can lead to liver cancer. The risk of HIV or hepatitis C virus transmission can basically be reduced by all forms of therapy for people with substance use disorder. Substitution treatments are the most successful in reducing intravenous drug use and the associated risks of infection.

By focusing overall attention on HIV, the risk of hepatitis has been massively underestimated in recent years: In prison, there are considerable risks for a spread of hepatitis that can already be described as “typical for prisons,” especially among intravenous drug users [56]. Chronic hepatitis B and C infections are associated with considerable mortality risks. As with HIV, the infection can be asymptomatic for a long time: it can take up to 20 years for liver failure to occur. Co-infections with HIV and hepatitis C, which are mainly found in people with substance use disorder, lead to even higher mortality and faster development of cirrhosis [57]. A secondary data analysis observed that OST, particularly when combined with other harm-reduction strategies, is an evidence-based measure of HIV and hepatitis C prevention, and that people who receive OST often show an increased compliance regarding antiretroviral treatment [58].

#### Further benefits of OST for people with opioid use disorder living in prison

Other arguments put forward for adequate substitution therapy in prison are as follows [12]: substance use disorder would persist, if untreated; the likelihood of committing other offences (e.g., possession of narcotics) and the promotion of drug trafficking in prison is higher without OST; high risks of infection due to the common use of nonsterile syringes when no sterile syringes are available officially. Moreover, substitution therapies are cost-effective and also much more cost-effective than other health care interventions, such as treatment of high blood pressure, HIV, or AIDS.

**Fig. 1:**
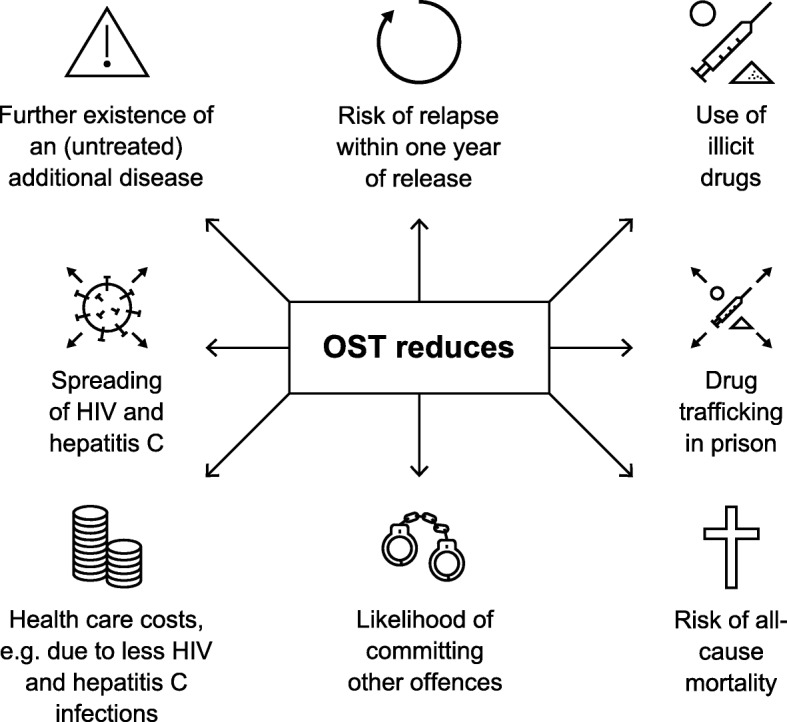
Reasons to use OST as standard in prisons and outside of prison

For the above-mentioned reasons (summarized in Fig. 1), the inequality between the treatment of people with opioid use disorder living in prison compared with people with opioid use disorder outside of prison should be unacceptable. A “cold withdrawal” or an insufficient medical accompaniment of a withdrawal is unlawful according to Article 3 of the ECHR. People living in prison have a right to the maximum attainable physical and mental health. Like people with diabetes or other chronic diseases, people receiving opioid agonist therapy depend on a daily medication to keep their disease in remission [59]. A refusal of OST in prisons leads to negative outcomes not only for the individuals but also for the institution, the communities, and society [1]. Opioid use disorder is a recognized chronic disease that requires treatment [60–62], and OST is a medical treatment—not a reward. This difference must be respected. Punishment consists of imprisonment and not the deprivation of fundamental human rights. In addition, we believe that people with opioid disorder living in prison should be given easily accessible psychosocial care, just as for people outside of prison for whom it contributes to the good results of opioid substitution.

### What people with opioid use disorder living in prison can do if OST is denied

People with opioid use disorder living in prison, who are denied OST, may appeal against the decision of the prison. Ready-made letters of complaint are available, for example, from Deutsche AIDS-Hilfe [12]. Since prison law does not require the use of a lawyer, people living in prison can formulate their own petitions/suits or ask for external assistance at the Chamber of Enforcement of Penalties. The courts often do not place too high demands on the formalities and contents. Only the deadlines have to be met and the purpose of the letter has to be made clear.

## Conclusion

The supply of OST inclusive psychosocial support in prison is still inadequate and falls short of the therapy standard for people outside of prison. There is still a gap between the number of people living in prison in need of substitution therapy and those receiving it, although benefits such as reduced risk of death in the post-release period, reduced risk of transmission of infectious diseases, and reduced reoffending have been shown. Physicians working within correctional facilities are caught in a “dual loyalty conflict” wherein the punitive aspect of the correctional facilities’ mission and the best interest of their patients often oppose each other [1]. It is important to free the physicians from this conflict and to accept that it will not be possible to solve a health problem by criminal law resources. It is also important to be aware that a substance use disorder is comparable with a chronic disease. No judge would prohibit a patient suffering from diabetes from using insulin and instead impose a diet and physical training on him because he does not believe in the effect of the approved drugs against diabetes. That would be ethically questionable. Reconsideration is urgently needed because people suffering from a disease and living in prison should be treated the same as people outside of prison and according to the latest state of medical research, and not be punished twice for their crimes and their illness. We believe the unequal treatment of patients in Germany in the different federal states and from prison to prison is irresponsible and discriminatory and prevents the achievement of resocialization.

Joint and coordinated efforts by the health system, the judicial system, and public health authorities are needed to ensure the best possible treatment of people living in prison and to minimize the adverse effects of illicit drug use. People living in prison who use illicit drugs should be informed by factsheets, videos, or discussion with health professionals about the services offered by the medical care and about strategies for obtaining the best possible treatment of their illness in prison and after release from prison.

## Data Availability

Not applicable

## Abbreviations

CNS: Central nervous system DGS German Society for Addiction Medicine ECHR European Convention on Human Rights FDA Food and Drug Administration OST Opioid substitution therapy UN United Nations UNODC United Nations Office on Drugs and Crime
